# Prevalence and risk factors of *Schistosoma mansoni* infection among children under two years of age in Mbita, Western Kenya

**DOI:** 10.1371/journal.pntd.0008473

**Published:** 2020-08-25

**Authors:** Miho Sassa, Evans A. Chadeka, Ngetich B. Cheruiyot, Mio Tanaka, Taeko Moriyasu, Satoshi Kaneko, Sammy M. Njenga, Sharon E. Cox, Shinjiro Hamano

**Affiliations:** 1 Department of Parasitology, Institute of Tropical Medicine (NEKKEN), Nagasaki University, Nagasaki, Japan; 2 School of Tropical Medicine and Global Health, Nagasaki University, Nagasaki, Japan; 3 The Joint Usage/ Research Centre on Tropical Diseases, Institute of Tropical Medicine (NEKKEN), Nagasaki University, Nagasaki, Japan; 4 Nagasaki University, Kenya Research Station, NUITM-KEMRI Project, Nairobi, Kenya; 5 Leading program, Program for Nuring Global Leaders in Tropical and Emerging Communicable Diseases, Graduate School of Biomedical Sciences, Nagasaki University, Nagasaki, Japan; 6 Department of Eco-Epidemiology, Institute of Tropical Medicine (NEKKEN), Nagasaki University, Nagasaki, Japan; 7 Eastern and Southern Africa Centre of International Parasite Control (ESACIPAC), Kenya Medical Research Institute (KEMRI), Nairobi, Kenya; 8 Department of Global Health Development Policy Science, Institute of Tropical Medicine (NEKKEN), Nagasaki University, Nagasaki, Japan; 9 Department of Population Health, London School of Hygiene & Tropical Medicine, London, United Kingdom; University of Nottingham, UNITED KINGDOM

## Abstract

Despite growing evidence that infants and very young children can be infected with schistosomes, the epidemiological features and risk factors are not well described in this age group. We aimed to assess the prevalence of *S*. *mansoni* infection in children under two years of age from a population with a known high burden of infection in school-aged children and adults and thus inform the need for interventions in this potentially vulnerable age group. In a cross-sectional study in Mbita Sub-county, along the east coast of Lake Victoria, Western Kenya, we enrolled 361 children aged 6–23 months. The prevalence of *S*. *mansoni* infection was detected using the Kato-Katz stool examination and a point-of-care test for urinary circulating cathodic antigen (POC-CCA) (Rapid Medical Diagnostics, Pretoria, South Africa). Three-hundred and five (305) children had complete data of whom 276 (90.5%, 95%CI: 86.6–93.5) children were positive for *S*. *mansoni* by the POC-CCA test, while 11 (3.6%, 95%CI: 1.8–6.4) were positive by the Kato-Katz method. All Kato-Katz positive cases were also positive by the POC-CCA test. In multivariable analysis, only geographical area, Rusinga West (AOR = 7.1, 95%CI: 1.4–35.2, P = 0.02), was associated with *S*. *mansoni* infection using Kato-Katz test. Independent associations for POC-CCA positivity included age, (12–17 months vs 6–11 months; AOR = 7.8, 95%CI: 1.8–32.6, P = 0.002) and breastfeeding in the previous 24 hours (AOR = 3.4, 95%CI: 1.3–9.0, P = 0.009).

We found a potentially very high prevalence of *S*. *mansoni* infection among children under two years of age based on POC-CCA test results in Mbita Sub-county, Kenya, which if confirmed strongly supports the need to include infants in public health strategies providing universal prophylactic treatment in high burden settings. Further research is required to determine the accuracy of diagnostic tools to detect light infection among very young children and possible long-term health impacts.

## Author summary

Schistosomiasis is one of the most common neglected tropical diseases (NTDs) worldwide with more than 200 million people at risk. Despite intensive control efforts and scaling up of programmes by public-private partnerships, schistosomiasis remains a serious public health problem. The true burden of schistosomiasis and its risk factors among very young children are not yet well-described. Here, we assessed schistosomiasis infection in 361 Kenyan children under two years of age in a high burden setting. We found extremely high prevalence of schistosomiasis (90.5%) when using a new, simple, rapid, point-of-care test which detects the presence of a schistosome glycoprotein (circulating cathodic antigen (CCA)) in urine samples, but a much lower prevalence (3.6%) when using the traditional method of stool microscopy for detecting the presence of schistosome eggs. Only area of residence was associated with schistosomiasis determined by Kato-Katz, whereas age and breastfeeding in the past 24 hours were independently associated with schistosomiasis by the point-of-care CCA test. Our findings reveal that very young children may carry an enormous burden of schistosomiasis and further research is required to determine how control programmes should encompass this neglected vulnerable group.

## Introduction

Schistosomiasis is a human parasitic disease predominant in tropical and subtropical areas with limited access to safe water and adequate sanitation. Globally, more than 200 million people, 111 million school-aged children (SAC) and 95 million adults, are estimated to be at risk of infection [1]. Schistosomiasis is classified as one of 20 neglected tropical diseases (NTDs) prioritized for a global response and targeted for elimination as a public health problem by 2025 [2]. There are two major forms of schistosomiasis of public health importance: intestinal and urogenital. Intestinal schistosomiasis is mainly caused by three species, *Schistosoma mansoni*, *Schistosoma japonicum*, and *Schistosoma mekongi* and primarily affects intestine and liver through acute and chronic inflammation against parasite eggs produced by adult worms [3–5].

Mass drug administration (MDA) with praziquantel for at-risk populations, particularly targeting school-aged children, has been the prime strategy used to control and eliminate schistosomiasis [6,7]. However, evidence is growing to support extension of MDA programmes to other age and population groups, including preschool-aged children [8–10].

Kenya carries substantial schistosomiasis burden mainly caused by two species, *S*. *mansoni* and *S*. *haematobium*, with more than 2.5 million people at risk of infection [11]. In line with the global strategy, an annual MDA programme, the Kenya national school-based deworming programme (NSBDP), was started in 2009, followed by a nation-wide scale up in 2012. In 2013, it was estimated that 0.9 million school-aged children in the endemic area were treated for schistosomiasis in Kenya [12].

Schistosomiasis severely affects children in most endemic settings, with negative impacts on health including undernutrition and growth retardation [13–17]. Much research has been done for school-aged children due to the epidemiological evidence that they are at the highest risk of infection and associated morbidity. However, more recently, growing evidence has emerged that preschool-aged children can also be infected with schistosomes [8,9,18].

The importance of health status during the “First 1,000 days” from conception to the second birthday of a child is emphasized as a critical development window in which interventions to improve health should be targeted as a means of improving long-term population health outcomes [19]. Prevention and treatment of schistosomiasis during this period may be an additional intervention to improve health and development. However, the true burden of schistosome infection in this critical period remains understudied. This lack of epidemiological evidence is in part due to operational difficulties of studies targeting young children including the limited ability to detect light infections, making it difficult to estimate the true extent of health impact among infants and young children [20]

To further enhance the progress of elimination efforts, it is likely to be necessary to address the disease burden among young children who are currently out of the target population for interventions. This study aimed to assess the prevalence of *S*. *mansoni* infection and its associated factors among this neglected, yet potentially vulnerable group in a high burden area and to compare the results using the recently developed point-of-care diagnostic detecting circulating cathodic antigen (CCA) with traditional methodology of Kato-Katz.

## Methodology

### Ethical consideration

The Scientific and Ethics Review Unit of the Kenya Medical Research Institute (SSC 2084, amendment 6) and The Ethical Committee of the Nagasaki University, Institute of Tropical Medicine (NEKKEN), Japan (No140829127-2) approved this study. Before the commencement of field activities, relevant information was provided to the Sub-county health offices and an authorization to conduct the survey was obtained.

All parents or guardians of participants were given written information about the study in their language of choice (English, Kiswahili or Dholuo) and advised that their participation was voluntary and that they could withdraw their consent at any time, without giving a reason and thereafter signed a consent form.

All children who were found positive for *S*. *mansoni* infection were treated with 40 mg/kg of praziquantel by a local clinician. Those who were infected with soil-transmitted helminths were treated with 200 mg of albendazole according to the WHO guideline [6]. Malaria positive children were also treated with artemether-lumefantrine. In addition, all anaemic children were provided with iron and folate tablets following national clinical guidelines [21].

### Study area

This study was conducted in Mbita, Homa Bay County, Western Kenya, located on the shore of Lake Victoria where intestinal schistosomiasis is known to be highly endemic [22,23]. The estimated population of Homa Bay County was 624,777 in 2019 [24], comprising mainly the Luo ethnic group. The area is inhabited by fishing and agricultural communities. According to the Kenya Demographic and Health Survey (KDHS) 2014, basic vaccination coverage (BCG, measles, DPT-HepB-Hib, polio, pneumococcal) was 64.4% in Homa Bay County. The prevalence of stunting, wasting, and underweight in the county among children under five years of age were 18.7%, 2.3%, and 5.4% [25].

**Fig 1:**
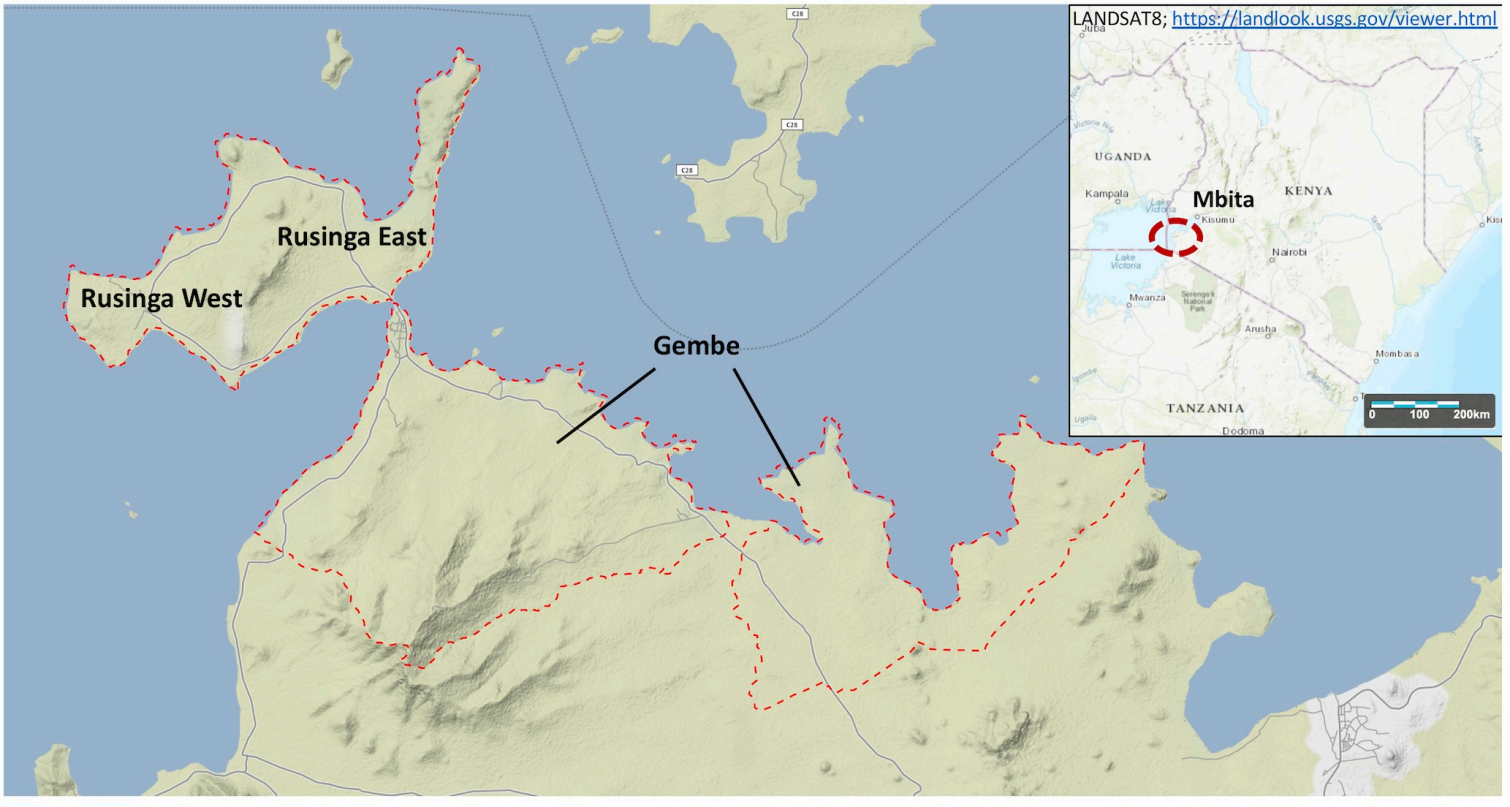
Study site in Mbita, Western Kenya.

A Health and Demographic Surveillance System (HDSS) has been established in Mbita since 2007, which provides a platform for population-based prospective studies [26]. The HDSS in Mbita covers three areas: Rusinga West, Rusinga East, and Gembe (**Fig 1**). The HDSS covered a population of 50,569 (24,432 males and 26,137 females) in 2010 across an area of 163.28 km^2^.

### Study, design, population, and sampling and recruitment strategies

This was an analytical cross-sectional study targeting children aged 6 to 23 months living in Mbita covered by the HDSS. This study employed two sampling strategies: (a) younger siblings of children aged 2–7 years being enrolled in a multi-parasite survey in early childhood development centres (ECDC) conducted in the same area [27] and; (b) simple random sampling of children 6 to 23 months of age from the Mbita HDSS. Trained research assistants conducted household visits of all eligible participants and obtained their informed consent from parents and/or legal guardians of the children.

### Questionnaire and anthropometric measurements

Study visits for data, anthropometry and specimen collection were conducted on designated days at public gathering places convenient to the study population. Trained research assistants conducted a structured interview using tablets and an ODK-based electronic questionnaire (https://getodk.org/) and conducted anthropometric measurements for all children. Weight was measured to the nearest 100 grams using TANITA BC-765 health scale (TANITA CO., Japan) and length was measured to the nearest mm by Seca 417 length scale (Seca Co., Germany). Mid-upper arm circumference (MUAC) was measured to the nearest mm by a MUAC tape provided by the local health office in Mbita Sub-county. HIV status of mother and child were confirmed from the mother and child health booklet.

### Sample collection and testing

After obtaining informed consent, the trained research assistants visited parents or main care-givers with pre-labelled specimen containers and paediatric urine collection bags and instructed them on their use for sample collection. Parents and/or care-givers were requested to take overnight stool and urine samples to the study data collection point the following morning. Infants arriving at the data collection point without urine samples had a new urine collection bag fitted by a research assistant and urine collected during the interview and clinical examination. Research assistants visited households of study participants up to three times to follow up if samples had not been received.

The Kato-Katz technique was applied for stool examination. Two Kato-Katz thick smear slides were prepared from each stool sample to detect parasite eggs [28]. The intensity of *S*. *mansoni* infection was expressed as the arithmetic mean number of eggs excreted per gram of faeces (epg) and categorised according to the WHO guideline [29]. Experienced technicians examined slides within one hour for hookworm eggs and later for schistosome, *Trichuris trichiura*, and *Ascaris lumbricoides* using a light microscope. For quality control, duplicated slides from the same stool were examined by different laboratory technicians. Urine samples were tested using a commercially available point-of-care circulating cathodic antigen (POC-CCA) test (Rapid Medical Diagnostics, Pretoria, South Africa) to detect *S*. *mansoni* antigen [30–32]. Test results were determined as negative, trace (barely visible result band), and positive based on the intensity of the result band. To address inter-observer discordance, interpretation of the POC-CCA test result was standardized as much as possible through the training of the technicians using written instructions and test results were determined by 2 independent observers, with any discordance resolved by a third reader.

Venous (3 ml) or capillary blood samples were collected after the interview by trained phlebotomists and tested for malaria using a rapid diagnostic test (*P*. *falciparum*, histidine-rich protein 2, CareStart, ACCESSBIO Co., USA), haemoglobin concentration (HemoCue, HemoCue AB Co., Angelhom, Sweden) and venous blood samples for a complete blood count (Xs-500i Sysmex Inc, Kobe, Japan).

### Sample size

Sample size calculations were performed to estimate the sample size required to determine the prevalence of *S*. *mansoni* infection as assessed by both Kato-Katz technique and by the more sensitive POC-CCA with a desired level of precision using the following formula:

$$n=\frac{1.96^{2}\times p(1-p)}{d^{2}}$$

Where *p* is an estimated proportion of interest and *d* is desired precision (one-sided width of 95% confidence interval). A sample size of 217 was calculated to be able to determine the prevalence of *S*. *mansoni* infection as assessed by Kato-Katz technique, estimated to be 17% based on a previous study among children of 12–24 months of age [33] with a desired absolute precision of at least +/-5%. Prevalence by POC-CCA was assumed to be at least two times greater than by Kato-Katz technique due to greater sensitivity and a sample size of 345 to determine prevalence of 34% with a desired absolute precision of at least +/-5% was calculated. Therefore, our planned target sample size was the larger of the two estimates.

### Data analysis

Data analysis was performed using STATA version 15 (Stata co., TX, USA). Anthropometric indicators, weight-for-length z-score (WLZ), length-for-age z-score (LAZ), and weight-for-age z-score (WAZ) were calculated using 2008 WHO reference population in STATA following WHO guidelines [34]. Based on these indices, binary indicators were set for underweight (WAZ < -2SD), stunting (LAZ < -2SD), and wasting (WLZ < -2SD). An index variable to represent household social economic status (SES) was created using principal components analysis (PCA) with the first component used as an index variable for SES categorized into tertiles (high, middle and low SES) for subsequent analysis [35]. Concordance between *S*. *mansoni* infection by Kato-Katz and POC-CCA (trace results treated as positive) tests was examined using the kappa index [36]. To describe the spatial distribution of *S*. *mansoni* infection, HDSS household coordinates were plotted using R version 3.6.1 [37] with nearest ECDC locations used for missing household location data. All maps were created using data by OpenStreetMap, under CC BY 3.0 (http://maps.stamen.com/#toner/12/37.7706/-122.3782).

To identify factors associated with *S*. *mansoni* infection, univariable and multivariable logistic regression methods were employed. Based on univariable analysis independent variables with a p-value of less than 0.2 for the crude odds ratio were included in multivariable logistic regression models together with age and sex as *a priori* variables. Variables were selected in a forward step-wise process and retained in the model if the p-value for the likelihood ratio test (LR test) comparing the models with and without the variable was less than 0.05.

## Results

### Characteristics of study participants

**Fig 2:**
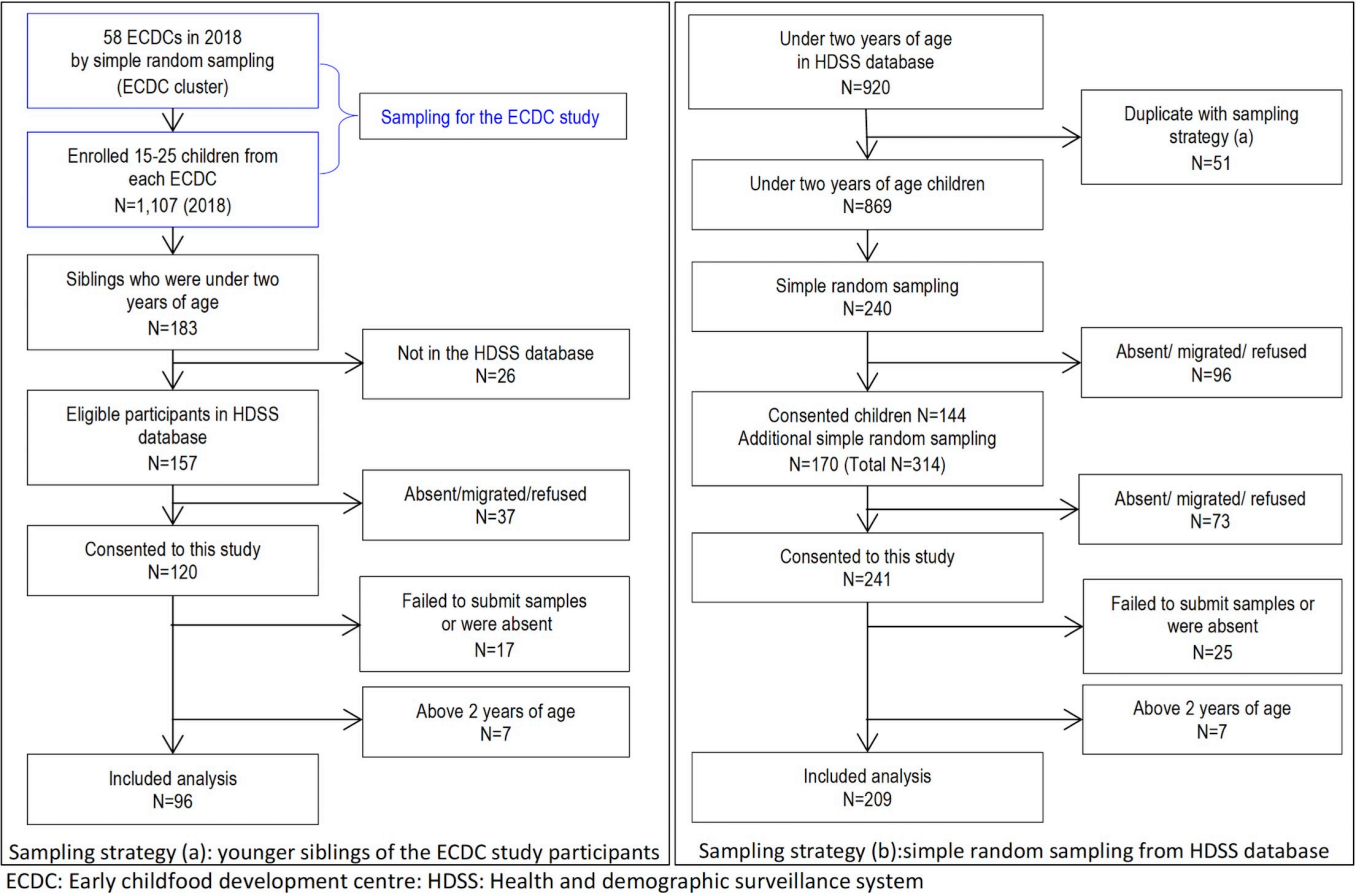
Sampling methodology.

This study enrolled 361 children aged from 6 to 23 months from 18 September to 26 November 2018. From 1,107 children enrolled in the ECDC study, 157 eligible younger siblings were identified, and consent obtained for 120 who were enrolled in the study (**Fig 2**). Of 920 potentially eligible children identified in the Mbita HDSS data, 410 children were selected by simple random sampling, of which 241 children were reached and agreed to participate (**Fig 2**). Forty-two children were later excluded because they did not submit urine or stool samples, or were absent during the clinical examination and interview and an additional 14 were excluded from data analysis because they were older than 2 years of age. When comparing the socio-demographic characteristics of the included and excluded participants, there were no statistical differences apart from age, sex, lake water contact in the past 7 days, and breastfed in the last 24 hours (**S1 Table**).

Of the 305 children included in this analysis (S1 Data), 156 were male (51.2%) and the mean age was 16.7 months (standard deviation; SD 4.6) with children enrolled through the HDSS sampling being significantly older (mean age: 14.7 vs 17.6 p<0.001, **Table 1**) but the two groups were similar in other respects apart from some differences in their fathers’ occupation (**Table 1**). Nearly half of the children were from Gembe in the mainland and the rest were from Rusinga Island. The majority of households (80.3%) used the lake water as the main source for drinking. 9.8% of study participants did not have a latrine in their house and practised open defecation. More than two-thirds of children (64.6%) had reported contact with lake water in the previous 7 days. The numbers of underweight children were 16 (5.2%). More than one-third of children (40.3%) were anaemic (<10 g/dl) [21]. The prevalence of malaria was 2.6%.

**Table 1:** Characteristics of study participants.

Variables	levels	ECDC siblings N (%)	HDSS N (%)	Total (%)	p-value
Overall		96	209		
Age (month)	Mean (SD)	14.7 (5.2)	17.6 (3.9)	16.7 (4.6)	<0.001
Sex	Male	51 (53.1)	105 (50.2)	156 (51.1)	0.64
Area of resident	Gembe	49 (51.0)	113 (54.1)	162 (53.1)	0.824
	Rusinga East	19 (19.8)	42 (20.1)	61 (20.0)	
	Rusinga West	28 (29.2)	54 (25.8)	82 (26.9)	
Education of mother	Primary	70 (72.9)	131 (62.7)	201 (65.9)	0.19
	Secondary	21 (21.9)	59 (28.2)	80 (26.2)	
	College/University	5 (5.2)	19 (9.1)	24 (7.9)	
Occupation of father	Business	13 (13.5)	41 (19.6)	54 (17.7)	0.039
	Farmer	4 (4.2)	6 (2.9)	10 (3.3)	
	Fishing	35 (36.5)	78 (37.3)	113 (37.0)	
	Petty trader	8 (8.3)	18 (8.6)	26 (8.5)	
	Office job/employed	25 (26.0)	24 (11.5)	49 (16.1)	
	Unemployed	5 (5.2)	21 (10.0)	26 (8.5)	
	No father	6 (6.2)	21 (10.0)	27 (8.9)	
Social Economic Status	Low	31 (32.3)	71 (34.0)	102 (33.4)	0.406
	Middle	37 (38.5)	65 (31.1)	102 (33.4)	
	High	28 (29.2)	73 (34.9)	101 (33.1)	
Water source for drinking	Lake water*^1^	75 (78.1)	170 (81.3)	245 (80.3)	0.512
Water source for bathing	Lake water*^1^	93 (96.9)	200 (95.7)	293 (96.1)	0.622
Water source for washing	Lake water*^1^	94 (97.9)	201 (96.2)	295 (96.7)	0.427
Toilet	Latrine	88 (91.7)	187 (89.5)	275 (90.2)	0.55
Lake water contact*^2^	Yes	63 (65.6)	134 (64.1)	197 (64.6)	0.798
Stunted*^3^	Yes	6 (6.2)	24 (11.5)	30 (9.8)	0.154
Underweight*^3^	Yes	5 (5.2)	11 (5.3)	16 (5.2)	0.984
Breastfed*^4^	Yes	54 (56.2)	109 (52.2)	163 (53.4)	0.505
Anaemia*^5^	Yes	34 (35.8)	88 (42.3)	122 (40.3)	0.283
Malaria (RDT)	Positive	3 (3.1)	5 (2.4)	8 (2.6)	0.71
HIV status of mother	Negative	78 (81.2)	171 (81.8)	249 (81.6)	0.834
	Positive	14 (14.6)	32 (15.3)	46 (15.1)	
	Unknown	4 (4.2)	6 (2.9)	10 (3.3)	
HIV status of child	Negative	90 (93.8)	189 (90.4)	279 (91.5)	0.578
	Positive	1 (1.0)	2 (1.0)	3 (1.0)	
	Unknown	5 (5.2)	18 (8.6)	23 (7.5)	

*1: vs Others

*2: Lake water contact in the past 7 days

*3: N = 289

*4: Breastfed in the last 24 hours

*5: N = 303, Anaemia: haemoglobin<10mg/dl

### Prevalence and distribution of *S*. *mansoni* infection by Kato-Katz and POC-CCA

Eleven children (3.6%; 95%CI: 1.8–6.4) were positive for the eggs of *S*. *mansoni* by Kato-Katz technique, while 276 children (90.5%; 95%CI: 86.6–93.5) were positive by the POC-CCA test (including ‘trace’). Only one child (0.3%) was infected with *A*. *lumbricoides*. None were infected with *T*. *trichiura*, or hookworm. All Kato-Katz positive samples were positive for the POC-CCA test. Among 294 samples which were negative by the Kato-Katz method, 265 were positive by the POC-CCA test. Only 29 children were negative by both methods. The agreement between the Kato-Katz and the POC-CCA test was very low with a kappa index of 0.0078 **(Table 2).**

**Table 2:** Concordance between Kato-Katz and POC-CCA (trace as positive) for *S*. *mansoni* infection.

		POC-CCA			Kappa index
		Positive	Negative	Total (%)	
**Kato-Katz**	Positive	11	0	11 (3.6)	0.0078
	Negative	265	29	294 (96.4)	
	Total (%)	276 (90.5)	29 (9.5)	305 (100)	

**Fig 3:**
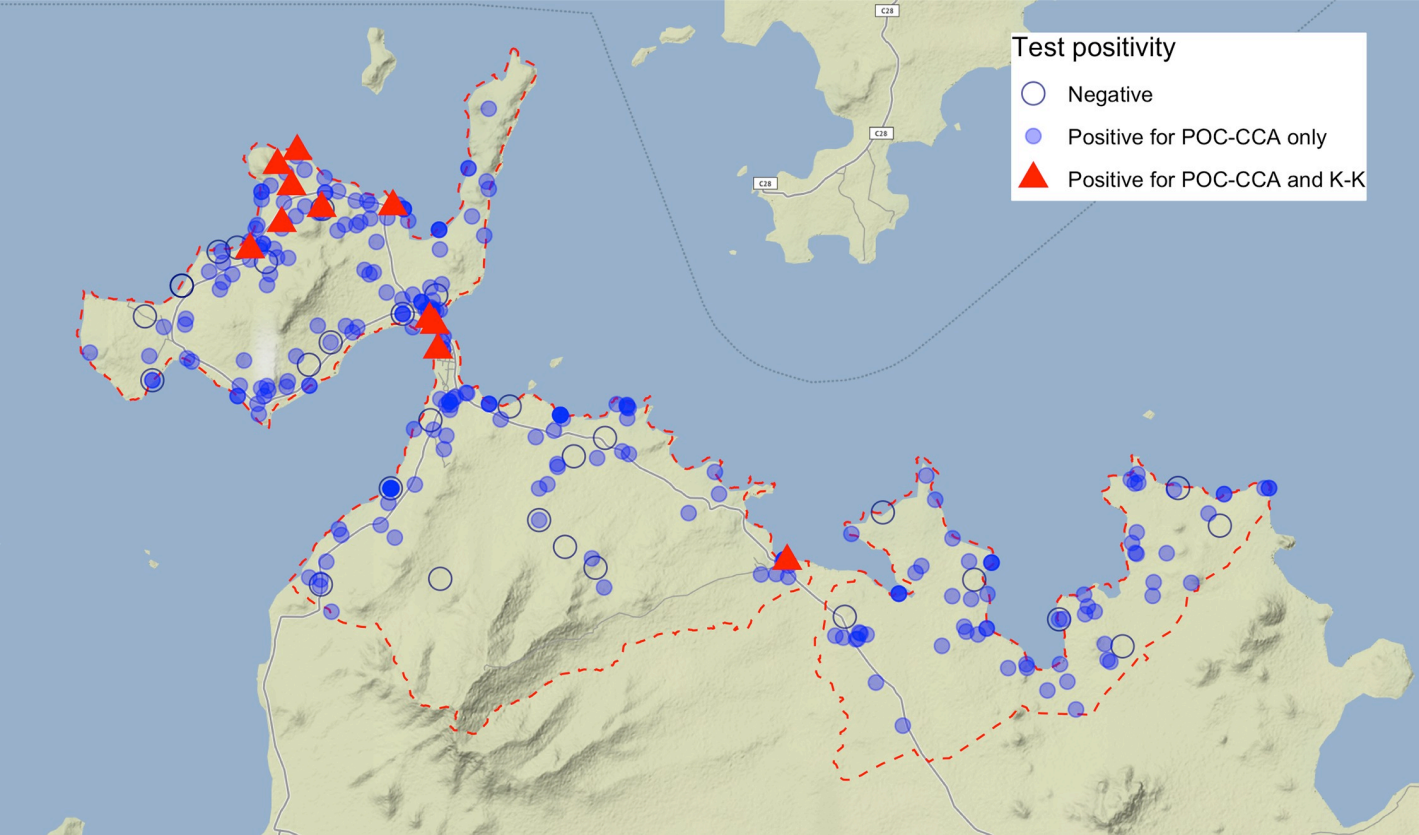
Spatial distribution of *S*. *mansoni* infection among children under two years of age in Mbita, Kenya.

**Fig 4:**
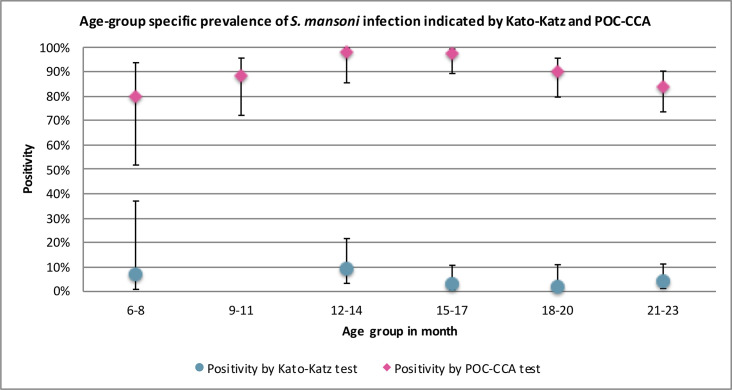
Age-group specific prevalence of *S*. *mansoni* infection determined by Kato-Katz and POC-CCA test.

Of 294 children who were *S*. *mansoni* negative by Kato-Katz, 198 were positive and 67 were trace positive by the POC-CCA test and one child with light *S*. *mansoni* infection by Kato-Katz was trace positive by POC-CCA (**Table 3**). The geographical distribution of *S*. *mansoni* infection by Kato-Katz and POC-CCA are shown in **Fig 3** with *S*. *mansoni* by Kato-Katz ranging from 1.2% in Gembe to 8.5% in Rusinga West (Fisher’s exact test p = 0.01 (**Table 4**). The majority of infected children harboured light infections (81.8%), while one child had a heavy infection (**Table 4**). Age-group-specific prevalence of *S*. *mansoni* infection determined by Kato-Katz and the POC-CCA tests are shown in **Fig 4**, with prevalence peaking in the 12–14 months age group for both tests. Of note, one child as young as 8 months of age was positive by Kato-Katz.

**Table 3:** Intensity of *S*. *mansoni* infection by Kato-Katz by POC-CCA category.

		POC-CCA*			Total (%)
		Negative	Trace	Positive	
Intensity by Kato-Katz	Negative	29	67	198	294 (96.4)
	Light	0	1	8	9 (3.0)
	Moderate	0	0	1	1 (0.3)
	Heavy	0	0	1	1 (0.3)
	Total (%)	29 (9.5)	68 (22.3)	208 (68.2)	305 (100)

* POC-CCA results

Negative: the control band developed, but no test band appeared

Trace: the band was barely visible

Positive: the test and control band appeared

**Table 4:** Intensity of the *S*. *mansoni* infection by Kato-Katz, by geographical area.

Infected (%)	Gembe	Rusinga East	Rusinga West	Total
	n=2/162 (1.2%)	n=2/61 (3.3%)	n=7/82 (8.5%)	n=11/305 (3.6%)
Light (1-99 epg*)	1 (50)	2 (100)	6 (85.7)	9 (81.8)
Moderate (100-399 epg*)	0	0	1 (14.3)	1 (9.1)
Heavy (>400 epg*)	1 (50)	0	0	1 (9.1)

*epg: arithmetic mean eggs per gram of faeces

### Risk factors of *S*. *mansoni* infection

The results of univariable and multivariable analysis of the association between *S*. *mansoni* infection confirmed by Kato-Katz or by POC-CCA and risk factors are shown in **Table 5**. Only geographical area was a significant risk factor for *S*. *mansoni* infection by Kato-Katz, with children resident in Rusinga West having increased odds of infection (AOR = 7.1, 95% CI: 1.4–35.2) compared to children resident in Gembe, adjusted for age and sex. For *S*. *mansoni* infection by POC-CCA in univariable analysis, age group (p = 0.002), breastfeeding within 24 hours prior to the interview (p = 0.003), using lake water for washing (p = 0.06), open defecation (p = 0.06), mother’s HIV status (p = 0.05), and child HIV status (p = 0.04) (not shown in table) showed possible evidence of association. However, in multivariable analysis, only age group; 12 to 17 months of age (AOR = 7.8, 95% CI: 1.8–32.6) compared to 6 to 11 months of age and breastfeeding within 24 hours prior to the interview (AOR = 3.4, 95% CI: 1.3–9.0) remained associated.

**Table 5:** Bivariable and multivariable analysis for *S*. *mansoni* infection by Kato-Katz and POC-CCA with crude and adjusted odds ratios (95%CI) for each potential risk factor.

Variables		Kato-Katz				POC-CCA			
		Univariable		Multivariable		Univariable		Multivariable	
		OR (95%CI)	p-value	AOR*^1^ (95%CI)	p-value*^2^	OR (95%CI)	p-value	AORAOR*^3^ (95%CI)	p-value*^4^
Age (Reference: 6-11 m)	12-17 months	2.6 (0.3–22.3)	0.50	2.3 (0.3–20.2)	0.66	6.3 (1.6–25.4)	0.002	7.8 (1.8–32.6)	0.002
	18-23 months	1.4 (0.2–12.9)		1.5 (0.2–14.5)		1.1 (0.4–2.7)		1.9 (0.7–5.5)	
Sex	Female	1.3 (0.4–4.2)	0.70	1.2 (0.3–4.0)	0.81	1.2 (0.6–2.6)	0.65	1.2 (0.5–2.6)	0.69
Area (Reference: Gembe)	Rusinga East	2.7 (0.4–19.7)	0.02	2.6 (0.4–19.5)	0.02	1.7 (0.5–5.2)	0.65		
	Rusinga West	7.5 (1.5–36.8)		7.1 (1.4–35.2)		1.1 (0.4–2.6)			
Mothers Education (Reference: Primary)	Secondary	3.3 (0.9–12.6)	0.12			1.8 (0.7–5.1)	0.45		
	College/University	4.5 (0.8–25.9)				1.4 (0.3–6.1)			
Father Occupation (Reference: Unemployed)	Business	1 (0.2–5.6)	0.48			4.6 (1.2–17.5)	0.15		
	Farmer	-				3.3 (0.4–31.2)			
	Fishing	0.3 (0.1–2.1)				3.8 (1.3–11.2)			
	Petty trader	-				9.2 (1–81.4)			
	Employed	0.5 (0.1–3.9)				3.2 (0.9–11.5)			
	No Father	-				9.6 (1.1–84.5)			
SES (Reference: Low)	Middle	3.1 (0.6–15.9)	0.31			1.6 (0.6–4.0)	0.62		
	High	1.5 (0.3–9.4)				1.4 (0.5–3.4)			
Water source for bath	Lake	0.4 (0.0–3.3)	0.44			3.4 (0.9–13.4)	0.11		
Water source for wash	Lake	0.3 (0.0–2.7)	0.36			4.4 (1.1–18.2)	0.06		
Toilet	Open defecation	-				0.4 (0.1–1)	0.06		
Water contact*^5^	Yes	1 (0.3–3.3)	0.95			0.8 (0.4–1.8)	0.60		
Urinate in lake*^5^	Yes	2.5 (0.5–12.5)	0.29			0.4 (0.2–1.3)	0.16		
Stunted	Yes	-				1.5 (0.3–6.7)	0.58		
Breastfeeding*^6^	Yes	1 (0.3–3.5)	0.94			3.4 (1.4–7.9)	0.003	3.4 (1.3–9.0)	0.009
Anaemia (<10g/dl)	Yes	0.5 (0.1–2.1)	0.36			0.7 (0.3–1.5)	0.36		
HIV of child (Reference: Negative)	Positive	-	0.85			0.05 (0.0–0.5)	0.04		
	Unknown	1.2 (0.1–10)				0.6 (0.2–2.3)			

Variables without statistically significant were omitted (Full table is available in **S2 and S3 Tables**).

*1 The adjusted odds ratios were based on the final logistic regression model with area, age and sex.

*2 Based on a likelihood ratio test. The variables were entered in the following order: areas, age and sex.

*3 The adjusted odds ratios were based on the final logistic regression model with age, breastfeeding, and sex.

*4 Based on a likelihood ratio test. The variables were entered in the following order: age, breastfeeding, and sex.

*5 in the past 7 days

*6 Breastfed in the last 24 hours

## Discussion

This study confirmed the existence of *S*. *mansoni* infection in young pre-school children in Mbita Sub-county, Western Kenya in ages as low as 8 months by Kato-Katz and a much higher prevalence by the more sensitive POC-CCA test in this high transmission area. There was no evidence of an association between *S*. *mansoni* infection and health status as assessed by nutritional status or anaemia in our study participants.

This study found an extremely high prevalence (90.5%) of *S*. *mansoni* infection determined by the POC-CCA test. In Western Kenya, Foo et al reported the prevalence of *S*. *mansoni* infection among children aged 8 to 12 years was 70.2% using the same POC-CCA test [38]. In a study in Cote d’Ivoire enrolling children from five months to five years of age, the prevalence determined by the same POC-CCA test was 80.0% with the earliest infection observed in a five-month-old child [39].

There was a large discordance between the Kato-Katz and the POC-CCA tests. Consistent with our study, previous studies comparing the two tests have generally showed similar discrepancies: 80.0% vs 21.9% by the POC-CCA and the Kato-Katz, among children aged 5 months to 5 years old in Cote d’Ivoire [39], and 62.1% vs 45.1% by the POC-CCA and Kato-Katz, also among preschool-aged children (aged below 6 years) in Uganda [40]. The latter study also showed the discrepancy of prevalence by the two tests was larger among children under 3 years of age than those in 3 to 5 years of age.

It is widely known that the Kato-Katz technique underestimates the prevalence of infection due to its low sensitivity, especially in settings where the prevalence and intensity of the infection are low [41–43]. A systematic review that examined the sensitivity of the POC-CCA test using the Kato-Katz test as a reference in different prevalence settings found that the relative sensitivity of the POC-CCA test tends to be higher in lower prevalence settings where infections are also generally of a lower intensity [44]. As younger children also tend to have a lighter intensity of infection compared to older children and adults [41,45], this might have contributed to the large discrepancy in prevalence by the two tests. In addition, the time-lag between schistosome infection to egg excretion into the stool, along with the schistosome life cycle and disease process [4] may also contribute to the higher prevalence by antigen detection from adult worms compared to egg detection in stools. Thus, the POC-CCA test is likely to better detect those with early-stage and low intensity infections.

However, there remains the possibility of false positive results in the POC-CCA test contributing to the very large differences in prevalence compared to by Kato-Katz. This could occur due to cross-reactivities of the POC-CCA test with other antigens or from maternal antigen transfer. It is reported in the kit insert [30] that urinary tract infections and haematuria may cause cross-reactivity of the test.

Although we did not actively look for potential factors that may cause such false-positivity, the strong independent association between breastfeeding in the preceding 24 hours and POC-CCA test positivity, adjusted for age and sex suggests this may be a possibility. Furthermore, whilst geographical area was the only factor associated with Kato-Katz positivity and is a good measure of the degree of exposure in older children in the study area [22], there was no indication of association with POC-CCA positivity. Finally, the positivity of POC-CCA test decreased in the oldest age group, when it might be expected to increase with increased exposure from toddling but is also when breastfeeding would become much less frequent. A previous study has demonstrated that *Schistosoma* antigen can pass to the fetus by placental transmission and to the breast milk in humans [46,47] but to our knowledge, cross-reactivity with this antigen has not been tested, nor if it can be found in infant urine.

It is widely known that geographical factors affecting degree of exposure are the most significant determinants of schistosomiasis in many settings. In our study, Gembe area had the lowest prevalence of *S*. *mansoni* infection determined by the Kato-Katz. The finding is in agreement with a previous study in the same area among school-aged children [22] which attributed higher prevalence in Rusinga Island compared to Gembe to higher population density. However, a more recent detailed study in these areas reported a very high degree of spatial heterogeneity in the prevalence and intensity of *S*. *mansoni* infection in preschool-aged children. [27] Further study is required to determine the basis of such heterogeneity including population densities of the intermediate host snail populations, not captured in the current studies.

We acknowledge several limitations of our study. Firstly, due to its cross-sectional design, this study could not determine the causality of observed associations and may not have been able to eliminate all potential confounders. Secondly, there is a possibility of bias due to a non-response rate of around 40% among those who were initially invited to the study. Thirdly, interpreting the result of the POC-CCA test might have caused an observer bias. When we performed the POC-CCA test in field conditions, the interpretation of a result by two observers often conflicted especially between “trace” and “negative”. To minimize this issue, the standard process was applied to determine test results as described in the methods section.

There is a growing awareness that children younger than school age can be infected by schistosomes with possible adverse health impacts [10,13,14,20,48–50] and hence they should be included in the treatment programme for schistosomiasis. However, children younger than school age have not been included in the schistosomiasis control programmes in most settings due to the lack of evidence on the magnitude of the prevalence and health impacts among them, and often limited resources. We believe our study adds valuable evidence to better understand the epidemiology of schistosomiasis among very young children and contribute to further policy discussions.

There is no established “gold standard” diagnostic method, particularly in the settings where the prevalence and intensity of infection are low. This study found many children who were schistosome egg-negative but antigen-positive identified by the POC-CCA test. Further research is needed to validate the accuracy of the POC-CCA by comparing with other diagnostic methods such as Enzyme-linked immunosorbent assay (ELISA) [51], circulating anodic antigen (CAA) test [52], or polymerase chain reaction (PCR) test [53], and investigating the possibility of antigen transfer.

The health impact of schistosome infection, particularly in children under two years of age, have been less studied than in older children [10,20]. Considering the chronic nature of the disease process, it may be necessary to conduct long-term follow-up studies to examine cumulative effects of schistosomiasis on child health, especially among those children who were exposed very early.

To conclude, we found a potentially very high prevalence of schistosomiasis among very young children in a highly endemic community in Western Kenya. Further research is required on accurate simple diagnostic tools to detect light infections among very young children and to determine the long-term health impacts of early infection, as well as effective public health strategies to address them. The evidence generated by further research should inform future schistosomiasis control policies to include young children who have attracted little attention to date.

## Supporting information

S1 Table Characteristics of the included and excluded children for analysis. *1: vs Others *2: Lake water contact in the past 7 days *3: N = 289, Underweight: WAZ<-2.0SD *4: Breastfed in the last 24 hours *5: N = 303, Anaemia: haemoglobin<10mg/dl.
(DOCX)

S2 Table Bivariable and multivariable analysis for *S*. *mansoni* infection by Kato-Katz test with crude and adjusted odds ratios (95%CI) for each potential risk factor. *1 The adjusted odds ratios were based on the final logistic regression model with area, age and sex. *2 Based on a likelihood ratio test. The variables were entered in the following order: areas, age and sex. *3 The adjusted odds ratios were based on the final logistic regression model with age, breastfeeding, and sex. *4 Based on a likelihood ratio test. The variables were entered in the following order: age, breastfeeding, and sex. *5: vs Others *6 in the past 7 days *7 Breastfed in the last 24 hours.
(DOCX)

S3 Table Bivariable and multivariable analysis for *S*. *mansoni* infection by POC-CCA test with crude and adjusted odds ratios (95%CI) for each potential risk factor. *1 The adjusted odds ratios were based on the final logistic regression model with area, age and sex. *2 Based on a likelihood ratio test. The variables were entered in the following order: areas, age and sex. *3 The adjusted odds ratios were based on the final logistic regression model with age, breastfeeding, and sex. *4 Based on a likelihood ratio test. The variables were entered in the following order: age, breastfeeding, and sex. *5: vs Others *6 in the past 7 days *7 Breastfed in the last 24 hours.
(DOCX)

S1 Data All data. (XLSX)
