# Supplementary material for: Prevalence and risk factors of Schistosoma mansoni infection among children under two years of age in Mbita, Western Kenya
Source: PLoS Negl Trop Dis. 2020 Aug 25;14(8):e0008473. doi: 10.1371/journal.pntd.0008473 (PMC7447014; doi:10.1371/journal.pntd.0008473)
Supplement: S1 Table — *1: vs Others *2: Lake water contact in the past 7 days *3: N = 289, Underweight: WAZ<-2.0SD *4: Breastfed in the last 24 hours *5: N = 303, Anaemia: haemoglobin<10mg/dl. (DOCX) [file pntd.0008473.s001.docx]

**S1 Table. Characteristics of the included and excluded children for analysis**

| **Variables** |  | **Included**  **N (%)** | **Excluded**  **N (%)** | **Total (%)** | **p-value** |
| --- | --- | --- | --- | --- | --- |
| Overall |  | 305 | 14 |  |  |
| Sex | Male | 156 (51.1) | 11 (78.6) | 167 (52.4) | 0.045 |
| Area of resident | Gembe | 162 (53.1) | 4 (28.6) | 166 (52.0) | 0.087 |
|  | Rusinga East | 61 (20.0) | 6 (42.9) | 67 (21.0) |  |
|  | Rusinga West | 82 (26.9) | 4 (28.6) | 86 (27.0) |  |
| Education of mother | Primary | 201 (65.9) | 8 (57.1) | 209 (65.5) | 0.65 |
|  | Secondary | 80 (26.2) | 4 (28.6) | 84 (26.3) |  |
|  | College/University | 24 (7.9) | 2 (14.3) | 26 (8.2) |  |
| Occupation of father | Buisiness | 54 (17.7) | 2 (14.3) | 56 (17.6) | 0.512 |
|  | Farmer | 10 (3.3) | 1 (7.1) | 11 (3.4) |  |
|  | Fishing | 113 (37.0) | 4 (28.6) | 117 (36.7) |  |
|  | Petty trader | 26 (8.5) | 1 (7.1) | 27 (8.5) |  |
|  | Employed | 49 (16.1) | 5 (35.7) | 54 (16.9) |  |
|  | Unemployed | 26 (8.5) | 0 (0.0) | 26 (8.2) |  |
|  | No father | 27 (8.9) | 1 (7.1) | 28 (8.8) |  |
| Social Economic Status | Low | 100 (32.8) | 7 (50.0) | 107 (33.5) | 0.388 |
|  | Middle | 102 (33.4) | 4 (28.6) | 106 (33.2) |  |
|  | High | 103 (33.8) | 3 (21.4) | 106 (33.2) |  |
| Waster source for drink | Lake water^*1^ | 245 (80.3) | 13 (92.9) | 258 (80.9) | 0.244 |
| Water source for bath | Lake water^*1^ | 293 (96.1) | 14 (100.0) | 307 (96.2) | 0.449 |
| Water source for wash | Lake water^*1^ | 295 (96.7) | 14 (100.0) | 309 (96.9) | 0.491 |
| Toilet | Laterine | 275 (90.2) | 13 (92.9) | 288 (90.3) | 0.739 |
| Lake water contact^*2^ | Yes | 197 (64.6) | 13 (92.9) | 210 (65.8) | 0.029 |
| Stunted^*3^ | Yes | 30 (9.8) | 3 (21.4) | 33 (10.3) | 0.164 |
| Underweight^*3^ | Yes | 16 (5.2) | 2 (14.3) | 18 (5.6) | 0.152 |
| Breastfed^*4^ | Yes | 163 (53.4) | 0 (0.0) | 163 (51.1) | <0.002 |
| Anaemia^*5^ | Yes | 122 (40.3) | 5 (35.7) | 127 (40.1) | 0.734 |
| Maralia result (RDT) | Yes | 8 (2.6) | 0 (0.0) | 8 (2.5) | 0.554 |
| HIV status of mother | Negative | 249 (81.6) | 12 (85.7) | 261 (81.8) | 0.554 |
|  | Positive | 46 (15.1) | 1 (7.1) | 47 (14.7) |  |
|  | Unknown | 10 (3.3) | 1 (7.1) | 11 (3.4) |  |
| HIV status of child | Negative | 279 (91.5) | 13 (92.9) | 292 (91.5) | 0.931 |
|  | Positive | 3 (1.0) | 0 (0.0) | 3 (0.9) |  |
|  | Unknown | 23 (7.5) | 1 (7.1) | 24 (7.5) |  |
| Kato-Katz result | Negative | 294 (96.4) | 13 (92.9) | 307 (96.2) | 0.497 |
|  | Positive | 11 (3.6) | 1 (7.1) | 12 (3.8) |  |
| POC-CCA result | Negative | 29 (9.5) | 2 (14.3) | 31 (9.7) | 0.555 |
| (trace as positive) | Positive | 276 (90.5) | 12 (85.7) | 288 (90.3) |  |

*1: vs Others *2: Lake water contact in the past 7 days *3: N=289 *4: Breastfed in the last 24 hours *5: N=303, Anaemia: haemoglobin<10mg/dl
