# Supplementary material for: Prevalence and risk factors of Schistosoma mansoni infection among children under two years of age in Mbita, Western Kenya
Source: PLoS Negl Trop Dis. 2020 Aug 25;14(8):e0008473. doi: 10.1371/journal.pntd.0008473 (PMC7447014; doi:10.1371/journal.pntd.0008473)
Supplement: S2 Table — *1 The adjusted odds ratios were based on the final logistic regression model with area, age and sex. *2 Based on a likelihood ratio test. The variables were entered in the following order: areas, age and sex. *3 The adjusted odds ratios were based on the final logistic regression model with age, breastfeeding, and sex. *4 Based on a likelihood ratio test. The variables were entered in the following order: age, breastfeeding, and sex. *5: vs Others *6 in the past 7 days *7 Breastfed in the last 24 hours. (DOCX) [file pntd.0008473.s002.docx]

**S2 Table. Bivariable and multivariable analysis for *S. mansoni* infection by Kato-Katz test with crude and adjusted odds ratios (95%CI) for each potential risk factor**

|  | |  | **Univariable** | | **Multivariable** | |
| --- | --- | --- | --- | --- | --- | --- |
| **Variables** | |  | **OR^*1^ (95%CI)** | **p-value^*2^** | **AOR^*3^ (95%CI)** | **p-value^*4^** |
| Age (Reference: 6-11M) | 12-17M | | 2.6 (0.3–22.3) | 0.50 | 2.3 (0.3–20.2) | 0.66 |
|  | 18-23M | | 1.4 (0.2–12.9) |  | 1.5 (0.2–14.5) |  |
| Sex | Female | | 1.3 (0.4–4.2) | 0.70 | 1.2 (0.3–4.0) | 0.81 |
| Area (Reference: Gembe) | Rusinga East | | 2.7 (0.4–19.7) | 0.02 | 2.6 (0.4–19.5) | 0.02 |
|  | Rusinga West | | 7.5 (1.5–36.8) |  | 7.1 (1.4–35.2) |  |
| Mothers Education (Reference: Primary) | Secondary | | 3.3 (0.9–12.6) | 0.12 |  |  |
|  | College/University | | 4.5 (0.8–25.9) |  |  |  |
| Father Education (Reference: Primary) | Secondary | | 1.3 (0.3–4.8) | 0.66 |  |  |
|  | College/University | | 2.3 (0.4–12.5) |  |  |  |
|  | No Father | | - |  |  |  |
| Mother Occupation (Reference: Housewife) | Business | | - | 0.83 |  |  |
|  | Farmer | | - |  |  |  |
|  | Fishing | | - |  |  |  |
|  | Petty trader | | 0.8 (0.2–3.5) |  |  |  |
|  | Employed | | 1.5 (0.3–8.0) |  |  |  |
| Father Occupation (Reference: Unemployed) | Business | | 1.0 (0.2–5.6) | 0.48 |  |  |
|  | Farmer | | - |  |  |  |
|  | Fishing | | 0.3 (0.1–2.1) |  |  |  |
|  | Petty trader | | - |  |  |  |
|  | Employed | | 0.5 (0.1–3.9) |  |  |  |
|  | No Father | | - |  |  |  |
| SES (Reference: Low) | Middle | | 3.1 (0.6–15.9) | 0.31 |  |  |
|  | High | | 1.5 (0.3–9.4) |  |  |  |
| Water source for drink | Lake^*5^ | | 1.1 (0.2–5.3) | 0.90 |  |  |
| Water source for bath | Lake^*5^ | | 0.4 (0.0–3.3) | 0.44 |  |  |
| Water source for wash | Lake^*5^ | | 0.3 (0.0–2.7) | 0.36 |  |  |
| Toilet | Open defecation | | - |  |  |  |
| Water contact^*6^ | Yes | | 1.0 (0.3–3.3) | 0.95 |  |  |
| Bathing in lake^*6^ | Yes | | 1.5 (0.5–5.1) | 0.49 |  |  |
| Playing in lake^*6^ | Yes | | 1.9 (0.6–6.8) | 0.32 |  |  |
| Urinate in lake^*6^ (Reference: No) | Yes | | 2.5 (0.5–12.5) | 0.29 |  |  |
|  | Don't know | | 5.5 (0.6–50.8) |  |  |  |
| Stunted | Yes | | - |  |  |  |
| Breastfeeding^*7^ | Yes | | 1.0 (0.3–3.5) | 0.94 |  |  |
| Anaemia | Yes | | 0.5 (0.1–2.1) | 0.36 |  |  |
| HIV of mother  (Reference: Negative) | Positive | | 0.5 (0.1–4.3) | 0.52 |  |  |
|  | Unknown | | - |  |  |  |
| HIV of child (Reference: Negative) | Positive | | - | 0.85 |  |  |
|  | Unknown | | 1.2 (0.1–10) |  |  |  |

*1 The adjusted odds ratios were based on the final logistic regression model with area, age and sex. *2 Based on a likelihood ratio test. The variables were entered in the following order: areas, age and sex. *3 The adjusted odds ratios were based on the final logistic regression model with age, breastfeeding, and sex. *4 Based on a likelihood ratio test. The variables were entered in the following order: age, breastfeeding, and sex. *5: vs Others *6 in the past 7 days *7 Breastfed in the last 24 hours
