# Supplementary material for: Prevalence and risk factors of Schistosoma mansoni infection among children under two years of age in Mbita, Western Kenya
Source: PLoS Negl Trop Dis. 2020 Aug 25;14(8):e0008473. doi: 10.1371/journal.pntd.0008473 (PMC7447014; doi:10.1371/journal.pntd.0008473)
Supplement: S3 Table — *1 The adjusted odds ratios were based on the final logistic regression model with area, age and sex. *2 Based on a likelihood ratio test. The variables were entered in the following order: areas, age and sex. *3 The adjusted odds ratios were based on the final logistic regression model with age, breastfeeding, and sex. *4 Based on a likelihood ratio test. The variables were entered in the following order: age, breastfeeding, and sex. *5: vs Others *6 in the past 7 days *7 Breastfed in the last 24 hours. (DOCX) [file pntd.0008473.s003.docx]

**S3 Table. Bivariable and multivariable analysis for *S. mansoni* infection by POC-CCA test with crude and adjusted odds ratios (95%CI) for each potential risk factor**

|  | |  | **Univariable** | | | **Multivariable** | | | | |
| --- | --- | --- | --- | --- | --- | --- | --- | --- | --- | --- |
| **Variables** | |  | **OR^*1^ (95%CI)** | **p-value^*2^** | | **OR^*3^ (95%CI)** | | **p-value^*4^** | | |
| Age (Reference: 6-11M) | 12-17M | | 6.3 (1.6–25.4) | | 0.002 | | 7.8 (1.8–32.6) | | 0.002 |  |
|  | 18-23M | | 1.1 (0.4–2.7) | |  |  | 1.9 (0.7–5.5) | |  |  |
| Sex | Female | | 1.2 (0.6–2.6) | | 0.65 | | 1.2 (0.5–2.6) | | 0.69 |  |
| Area (Reference: Gembe) | Rusinga East | | 1.7 (0.5–5.2) | | 0.65 | |  | |  |  |
|  | Rusinga West | | 1.1 (0.4–2.6) | |  |  |  | |  |  |
| Mothers Education (Reference: Primary) | Secondary | | 1.8 (0.7–5.1) | | 0.45 | |  | |  |  |
|  | College/University | | 1.4 (0.3–6.1) | |  |  |  | |  |  |
| Father Education (Reference: Primary) | Secondary | | 1.0 (0.5–2.4) | | 0.78 | |  | |  |  |
|  | College/University | | 1.5 (0.3–6.9) | |  |  |  | |  |  |
|  | No Father | | 2.4 (0.3–19.2) | |  |  |  | |  |  |
| Mother Occupation (Reference: Housewife) | Business | | 0.5 (0.2–1.3) | | 0.30 | |  | |  |  |
|  | Farmer | | 0.6 (0.1–3.1) | |  |  |  | |  |  |
|  | Fishing | | - | |  |  |  | |  |  |
|  | Petty trader | | 1.7 (0.5–5.5) | |  |  |  | |  |  |
|  | Employed | | 1.3 (0.3–6.1) | |  |  |  | |  |  |
| Father Occupation (Reference: Unemployed) | Business | | 4.6 (1.2–17.5) | | 0.15 | |  | |  |  |
|  | Farmer | | 3.3 (0.4–31.2) | |  |  |  | |  |  |
|  | Fishing | | 3.8 (1.3–11.2) | |  |  |  | |  |  |
|  | Petty trader | | 9.2 (1.0–81.4) | |  |  |  | |  |  |
|  | Employed | | 3.2 (0.9–11.5) | |  |  |  | |  |  |
|  | No Father | | 9.6 (1.1–84.5) | |  |  |  | |  |  |
| SES (Reference: Low) | Middle | | 1.6 (0.6–4) | | 0.62 | |  | |  |  |
|  | High | | 1.4 (0.5–3.4) | |  |  |  | |  |  |
| Water source for drink | Lake^*5^ | | 1.3 (0.5–3.3) | | 0.53 | |  | |  |  |
| Water source for bath | Lake^*5^ | | 3.4 (0.9–13.4) | | 0.11 | |  | |  |  |
| Water source for wash | Lake^*5^ | | 4.4 (1.1–18.2) | | 0.06 | |  | |  |  |
| Toilet | Open defecation | | 0.4 (0.1–1.0) | | 0.06 | |  | |  |  |
| Water contact^*6^ | Yes | | 0.8 (0.4–1.8) | | 0.60 | |  | |  |  |
| Bathing in lake^*6^ | Yes | | 1.3 (0.6–2.9) | | 0.47 | |  | |  |  |
| Playing in lake^*6^ | Yes | | 0.9 (0.4–2.3) | | 0.91 | |  | |  |  |
| Urinate in lake^*6^ (Reference: No) | Yes | | 0.4 (0.2–1.3) | | 0.16 | |  | |  |  |
|  | Don't know | | - | |  |  |  | |  |  |
| Stunted | Yes | | 1.5 (0.3–6.7) | | 0.58 | |  | |  |  |
| Breastfeeding^*7^ | Yes | | 3.4 (1.4–7.9) | | 0.003 | | 3.4 (1.3–9.0) | | 0.009 |  |
| Anaemia (combined) | Yes | | 0.7 (0.3–1.5) | | 0.36 | |  | |  |  |
| HIV of mother  (Reference: Negative) | Positive | | 0.5 (0.2–1.2) | | 0.05 | |  | |  |  |
|  | Unknown | | 0.2 (0.0–0.8) | |  |  |  | |  |  |
| HIV of child (Reference: Negative) | Positive | | 0.05 (0.0–0.5) | | 0.04 | |  | |  |  |
|  | Unknown | | 0.6 (0.2–2.3) | |  |  |  | |  |  |

*1 The adjusted odds ratios were based on the final logistic regression model with area, age and sex. *2 Based on a likelihood ratio test. The variables were entered in the following order: areas, age and sex. *3 The adjusted odds ratios were based on the final logistic regression model with age, breastfeeding, and sex. *4 Based on a likelihood ratio test. The variables were entered in the following order: age, breastfeeding, and sex. *5: vs Others *6 in the past 7 days *7 Breastfed in the last 24 hours
